# Financial literacy, behavioral traits, and ePayment adoption and usage in Japan

**DOI:** 10.1186/s40854-023-00504-3

**Published:** 2023-06-11

**Authors:** Trinh Quang Long, Peter J. Morgan, Naoyuki Yoshino

**Affiliations:** 1 Faculty of Business, FPT University HCM Campus, Ho Chi Minh City, Vietnam; 2 Senior Consulting Economist and Advisor to the Dean, Asian Development Bank Institute, Kasumigaseki 3-2-5, Chiyoda-ku, Tokyo, 100-6008 Japan; 3 Faculty of Economics, Keio University, 2 Chome-15-45 Mita, Minato City, Tokyo, 108-8345 Japan

**Keywords:** Financial literacy, Financial literacy heterogeneity, Herd behavior, Risk aversion, ePayment adoption, ePayment usage, Electronic money, Mobile payment app, Japan, D14, G11, J26

## Abstract

This study investigates how financial literacy and behavioral traits affect the adoption of electronic payment (ePayment) services in Japan. We construct a financial literacy index using a representative sample of 25,000 individuals from the Bank of Japan’s 2019 Financial Literacy Survey. We then analyze the relationship between this index and the extensive and intensive usage of two types of payment services: electronic money (e-money) and mobile payment apps. Using an instrumental variable approach, we find that higher financial literacy is positively associated with a higher likelihood of adopting ePayment services. The empirical results suggest that individuals with higher financial literacy use payment services more frequently. We also find that risk-averse people are less likely to adopt and use ePayment services, whereas people with herd behavior tend to adopt and use ePayment services more. Our empirical results also suggest that the effects of financial literacy on the adoption and use of ePayment differ among people with different behavioral traits.

**Supplementary Information:**

The online version contains supplementary material available at 10.1186/s40854-023-00504-3.

## Introduction

In recent decades, the progress of financial technology (fintech) has helped consumers access financial markets and services more easily than before (Kou et al. 2021). As a result, the number of financial products offered has increased, and simultaneously, such products have become more complicated. The literature shows that adopting fintech services helps increase financial inclusion and improve consumers’ financial well-being (Suri 2017). Among fintech services, consumers, firms, and governments commonly use electronic payment.^1^ Adopting ePayment services helps develop a cashless economy, reduces business costs for all parties, and increases economic efficiency (Rogoff and Rogoff 2017; Xu et al. 2019). Using ePayment services helps reduce the estimated gross domestic product (GDP) losses due to the use of cash: 0.12% of the GDP in Germany (Cabinakova et al. 2017); 0.45% of the GDP in Canada (Kosse et al. 2017); 0.29% of the GDP in Japan (Fujiki 2022).

While fintech services play an increasingly important role in individuals’ financial activities, they entail numerous risks, including both traditional financial risks and new Internet-related risks (Li et al. 2021; Morgan et al. 2019). Thus, to overcome these risks, consumers should have adequate knowledge to make financial decisions about adopting and using fintech services. However, among the various studies examining the factors that affect the adoption of ePayment services (Jack et al. 2013; Afawubo et al. 2020), only a few have explicitly investigated the role of financial literacy in the adoption of fintech products (Li et al. 2020; Morgan and Long 2018; Foster and Johansyah 2021). Moreover, these studies provide mixed empirical results on the role of financial literacy in ePayment and fintech adoption, while others conclude that financial literacy is positively associated with fintech/ePayment adoption (Morgan and Long 2018; Foster and Johansyah 2021), while others find a negative relationship (Li et al. 2020). In addition, previous studies show that the effect of financial literacy on financial decisions may vary by sex, rural–urban location, or education level (Xu et al. 2022). Moreover, the relationship between behavioral traits such as risk aversion or herd behavior and the heterogeneity of the effects of financial literacy on fintech adoption are yet to be studied. Therefore, answering questions such as whether the effect of financial literacy differs for individuals with different behavioral traits may provide empirical evidence to help develop targeted financial literacy programs.

This study attempts to fill these gaps in the literature by examining the effects of financial literacy on the adoption and use of ePayment services in Japan. First, we construct a financial literacy index that reflects financial knowledge, financial behavior, financial attitudes, and knowledge of the practical management of financial assets. We then investigate the relationship between this financial literacy index and the extensive and intensive use of two types of payment services: chip-based electronic money (e-money)^2^ and mobile payment apps.^3^ We also examine how two behavioral traits (herd behavior and risk aversion) may affect the relationship between the financial literacy index and the adoption and usage of ePayment services. Finally, we use the instrumental variable (IV) approach to address the potential endogeneity of financial literacy. The Bank of Japan collected the dataset in 2019, with a sample size of 25,000 adults. The sample was designed to represent the adult Japanese population.

Our empirical results show that higher financial literacy is positively associated with a higher likelihood of adopting and using both ePayment services. The results also suggest that risk aversion is negatively associated with the adoption and usage of ePayment services, whereas herd behavior is positively correlated with ePayment adoption and use. We also find that the effects of financial literacy on the adoption and usage frequency of ePayment services differ between individuals with and without risk aversion. For those with herd behavior, higher financial literacy tends to be associated with a lower adoption rate of mobile payment apps but does not affect e-money adoption.

This study extends the existing literature in several ways. First, it is related to the growing literature on the role of financial literacy in adopting and using fintech services. To the best of our knowledge, few studies have examined the effect of financial literacy (or financial knowledge) on the adoption and use of fintech services (Li et al. 2020; Morgan and Long 2018; Foster and Johansyah 2021), and the results are mixed. For example, Morgan and Long (2018) find that financial literacy positively correlates with awareness of fintech services in Lao PDR. In addition, Foster and Johansyah (2021) find that financial literacy positively correlates with adopting chip-based e-money in Indonesia. By contrast, Li et al. (2020) find a negative relationship between financial knowledge and ePayment adoption in the US. Our study provides further evidence of the effects of financial literacy on the adoption and intensity of fintech use.

Second, this study contributes to the literature on consumer adoption of technology. While previous literature has used several theories of technology adoption, such as the Diffusion of Innovation (DOI), Technological Adoption Model (TAM), and Unified Theory of Acceptance and Usage of Technology (UTAUT2), none of these models explicitly consider the role of financial literacy and behavioral traits.^4^ Moreover, only a limited number of empirical studies have examined how these two factors affect the adoption of fintech products (Li et al. 2020). Our study adds to the literature by examining the effects of financial literacy, risk aversion, and herd behavior in Japan.

Third, our study contributes to the literature on the heterogeneity of the effects of financial literacy. Specifically, we investigate whether financial literacy affects individuals with different behavioral traits. While previous literature has demonstrated that behavioral traits and financial literacy play important roles in individuals’ financial decisions (Almenberg and Dreber 2015; Gathergood and Weber 2017; Grohmann 2018; Hsiao and Tsai 2018; Van Rooij et al. 2011), only a few studies have examined the heterogeneity of the effect of financial literacy across individuals with different behavioral traits. For example, Jiang et al. (2020) show that the effect of financial literacy on financial well-being is greater among risk-averse individuals. This study extends this strand of the literature by examining how behavioral traits may affect the relationship between financial literacy and fintech adoption and use.

Japan is an interesting case study to examine the role of financial literacy and fintech adoption. On the one hand, as a highly developed economy, Japan has adequate foundations (in terms of financial regulation, financial structure, and technical knowledge) and policies (e.g., the Japanese government’s recent policy to subsidize cashless payments) to promote the use of fintech (Fahey 2019). On the other hand, the adoption of fintech is limited, especially compared to China and South Korea. Ernst and Young (2019) show that the fintech adoption rate in Japan is low (approximately 34% in 2019 vs. 87% in China, 67% in South Korea, and 46% in the US). Moreover, the gap in the adoption rate between Japan and the global average widened from 19 percentage points in 2017 to 26 percentage points in 2019 (Ernst and Young 2019). According to the Central Council for Financial Services Information (CCFSI; 2016), the financial literacy of the Japanese population is slightly lower than that of Americans, Germans, and the British. Therefore, researchers and policymakers want to understand the relationship between financial literacy and ePayment adoption in Japan.

The remainder of this paper is organized as follows. First, we briefly review the literature and propose testable hypotheses. "Empirical approach" section presents the data, empirical approach, and descriptive data. "Estimation results" section presents the empirical results. Finally, "Discussion and concluding remarks" section discusses the results and their theoretical, practical, and policy implications.

## Literature review and hypothesis development

### Financial literacy and ePayment adoption

Previous studies have treated fintech services as technological innovations and analyzed their adoption using various theories, including the DOI theory (Rogers 2003), Initial Trust Model (Kim and Prabhakar 2004), TAM (Davis 1989), UTAUT Model (Venkatesh et al. 2003), and UTAUT2 Model (Venkatesh et al. 2012). Additionally, previous studies have examined other factors that explain the usage of fintech services. These studies show that an individual’s perceived usefulness and ease of use, performance expectancy, perceived risk, perceived trust, and facilitation conditions are major determinants of fintech service adoption (e.g., Laukkanen and Pasanen 2008; Baptista and Oliveira 2016; Malaquias and Hwang 2016).

Financial literacy may also affect the adoption and use of ePayment services. Given the increasing responsibilities that consumers need to assume in planning for retirement and using credit, there is an increasing focus on whether consumers are sufficiently well-equipped to deal with financial matters. Financial literacy is the knowledge and understanding of financial concepts used to make effective financial choices.^5^ Lusardi et al. (2017) develop a theoretically augmented stochastic life cycle model that endogenizes the decision to acquire financial literacy. The model predicts that different levels of financial literacy account for sizable differences in wealth holdings across education groups. Many studies have empirically shown a strong correlation between financial literacy and financial behavior, such as daily financial management skills, participation in financial markets, investing in stocks, and engaging in precautionary savings (Hilgert et al. 2003; Christelis et al. 2010; van Rooij et al. 2011; de Bassa Scheresberg 2013; Yoshino et al. 2017). Research has corroborated these results in countries, such as Japan (Yoshino et al. 2017), Cambodia, Laos, Vietnam (Morgan and Long 2019, 2020), and Bangladesh (Hasan et al. 2021).

Research suggests that high financial literacy motivates individuals to process information, set up a business, acquire new financial knowledge, and search for what is available in the market. These characteristics pave the way for the adoption of new services such as fintech. Financial literacy affects the adoption of fintech services through two channels. First, higher financial literacy lowers the information costs incurred owing to the use of a new financial product. The literature shows that the likelihood of using a financial product, especially a risky one, is crucially affected by the costs and benefits of acquiring information (Hsiao and Tsai 2018). Therefore, lowering the cost of acquiring such information influences the decision to use that product (Vissing-Jorgensen 2003; Guiso and Jappelli 2005). Second, financial literacy may help mitigate a customer’s risks when using fintech services. Individuals with higher financial literacy are more likely to avoid such risks because they have a higher propensity to choose suitable financial products (Agarwal et al. 2020; Gathergood and Weber 2017), detect risky services (Engels et al. 2020), and detect fraud (Wei et al. 2021). Several studies have examined the effects of financial literacy on the adoption of fintech services, including ePayment services (Li et al. 2020; Morgan and Long 2018; Foster and Johansyah 2021; Lo Prete 2022), mobile money, digital banking (Yates 2020; Frimpong et al. 2022; Chen and Xiang 2021), peer-to-peer (P2P) lending (Gonzalez 2022), or holding cryptocurrencies (Fujiki 2020). While some studies argue that individuals with higher financial literacy might recognize the risks they may incur when using fintech services, they are less likely to adopt fintech services (Li et al. 2020; Chen and Xiang 2021). In contrast, most studies show a positive correlation between financial literacy and the adoption and usage of fintech services. Based on the above analysis, we propose the following hypothesis:

#### H1

Individuals with a higher level of financial literacy are more likely to adopt and use fintech services than those with a lower level of financial literacy.

### Financial literacy, behavioral traits, and ePayment adoption and use

The literature shows that behavioral traits play an important role in individuals’ financial decisions. Risk attitude is an essential factor influencing various personal financial decisions (Snelbecker et al. 1990). Risk attitudes are important in financial planning models and consumer decision-making frameworks (Han et al. 2019). Ajzen and Fishbein (1975) posit that risk attitudes affect individuals’ beliefs and, ultimately, influence their decisions. Therefore, individuals with different levels of risk aversion may exhibit different financial investment behaviors. Previous studies have shown that risk preferences can influence risky financial decisions, such as participation in stock markets or holdings of risky assets (Badarinza et al. 2016; Barberis et al. 2006). Therefore, risk preferences may explain the differences in the uptake of fintech services among individuals. Lin et al. (2013) examine the online P2P lending market and find a significant positive correlation between risk-loving attitudes and Internet financing volume. Similarly, Han et al. (2019) show that financial knowledge and risk attitudes are strongly associated with participation in P2P lending in China.

Among risk-averse individuals, those with a higher level of financial literacy may not be as affected by risk aversion in their decision to adopt fintech services compared with those with a lower level of financial literacy. Thus, the effect of financial literacy may be greater among risk-averse individuals. Morgan et al. (2019) argue that fintech services entail numerous risks that are more diverse and harder to spot than those associated with traditional financial products and services. Studies have shown that education may encourage risk-averse individuals to take risks (Dohmen et al. 2005; Hryshko et al. 2011). Moreover, Jung (2015) argues that higher education might diminish risk aversion through a better understanding of how to deal with risks. Therefore, we propose the following four hypotheses.

#### H2a

The effect of financial literacy on the adoption of e-money is higher for risk-averse people.

#### H2b

The effect of financial literacy on the adoption of mobile payment apps is higher for risk-averse people.

#### H2c

The effect of financial literacy on the usage frequency of e-money is higher for risk-averse people.

#### H2d

The effect of financial literacy on the usage frequency of mobile payment apps is higher for risk-averse people.

Herd behavior is another trait widely studied in financial markets, especially in stock markets. Zhang and Chen (2017) define herding as “individuals doing what other individuals are doing, even when their information suggests them to do something different from the others.” Individuals with herd behavior may ignore their viewpoints and expertise, regardless of whether they are valid, to make decisions consistent with the herd (Devenow and Welch 1996). In such cases, failure is the result of a mistake made by the herd rather than one of the herd members (Ahmad and Mahmood 2020). The literature shows herd effects are strong determinants of portfolio choice and stock market participation (Hong et al. 2004; Brown et al. 2008; Van Rooij et al. 2011). Since individuals with herd behavior tend to ignore their expertise and judgment and follow the herd in making decisions, the effect of financial literacy on the adoption and usage of fintech services may differ for individuals with herd behavior, depending on the herd’s decisions. Widely used fintech services (e.g., e-money) may be adopted by individuals with herd behavior to the same extent as those without herd behavior. Early adopters can obtain tips, advice, or information about fintech services. Thus, the effect of financial literacy may not differ between individuals with and without herd behavior. However, for new services only adopted by a limited number of people (e.g., mobile payment apps),^6^ the adoption is likely to be lower for those with herd behavior. Regarding usage frequency, whether the effect of financial literacy differs between individuals with and without herd behavior may depend on the herd size. If a herd is large, financial literacy may not vary significantly. Therefore, we propose the following hypotheses:

#### H3a

The effect of financial literacy on the adoption of e-money is not different between individuals with and without herd behavior.

#### H3b

The effect of financial literacy on the adoption of mobile payment apps is lower among individuals with herd behavior.

#### H3c

The effect of financial literacy on the usage frequency of e-money is not different among individuals with herd behavior.

#### H3d

The effect of financial literacy on the usage frequency of mobile payment apps is lower among individuals with herd behavior.

## Empirical approach

### Data source

The Bank of Japan’s Financial Literacy Survey is an online questionnaire survey conducted to understand the current state of financial literacy, that is, the financial knowledge and financial decision-making skills of individuals aged 18–79 years in Japan, chosen in proportion to Japan’s current demographic structure (CCFSI 2016, 2019). The first survey was conducted in 2011 by the CCFSI, followed by the second and third rounds in 2016 and 2019, respectively. Twenty-five thousand individuals participated in the 2016 and 2019 surveys. This study uses data from the 2019 survey because information on fintech usage was available only in this survey.

Questions on financial literacy include true/false questions on “financial knowledge and financial decision-making skills” and “characteristics of behavior and attitude.” Approximately half of the questions are similar to those in the surveys conducted by the U.S. Financial Industry Regulatory Authority Investor Education Foundation and the Organization for Economic Co-operation and Development (OECD; CCFSI 2016, 2019). Information on sex, age, place of residence, occupation, annual income, and experience of participating in financial education is collected. Finally, information on using fintech services and products is also collected.

### Empirical approach

To quantify the effect of financial literacy on the decision to adopt ePayment services, we estimate the following equation:

1 $$F{T}_{i}={\beta }_{0}+{\beta }_{1}F{L}_{i}+{\beta }_{2}Be{have}_{i}+{\beta }_{3}{X}_{i}+{\eta }_{i},$$

where the dependent variable $F{T}_{i}$ indicates whether individual $i$ uses an ePayment service (e-money or mobile payment app). $F{L}_{i}$ is the financial literacy index value of individual $i$; $Be{have}_{i}$ is a set of two behavioral traits (risk-averse or herd behavior) of individual $i$; ${X}_{i}$ is a vector of the control variables; and ${\eta }_{i}$ is the error term. We use linear probability regression to estimate Eq. (1) because we use the IV approach, and the linear probability regression allows us to test the validity of the IVs in a straightforward manner.

We also analyze how financial literacy affects the usage frequency of e-money or mobile payment apps. As described below, because our dependent variable depicts the ordering of the usage frequency, we estimate an ordered probit model in which the dependent variable takes one of the four intensity levels. We assume the existence of a latent continuous exact variable $F{T}_{i}^{*}$ that determines the order of the intensity of using ePayment services. The following equation characterizes the underlying:

2 $$F{T}_{i}^{*}={\alpha }_{0}+{\alpha }_{1}F{L}_{i}+{\alpha }_{2}Behav{e}_{i}+{\alpha }_{3}{X}_{i}+{\epsilon }_{i},$$

where $F{T}_{i}^{*}$ is the observed category of a response corresponding to the ith order of ePayment usage intensity, $F{L}_{i}$ is the financial literacy index, $Be{have}_{i}$ is a set of two behavioral traits of individual $i$, ${and X}_{i}$ is a vector of the control variables. All the independent variables in this equation are the same as those in Eq. (1).

We further examine whether financial literacy mitigates behavioral traits by augmenting Eqs. (1) and (2) as follows:

3 $$F{T}_{i}={\gamma }_{0}+{\gamma }_{1}F{L}_{i}+{\gamma }_{2}Behav{e}_{i}+{\gamma }_{3}Behav{e}_{i}*F{L}_{i}+{\gamma }_{4}{X}_{i}+{\varepsilon }_{i}$$

and

4 $$F{T}_{i}^{*}={\theta }_{0}+{\theta }_{1}F{L}_{i}+{\theta }_{2}{Behave}_{i}+{\theta }_{3}{Behave}_{i}*{FL}_{i}+{\theta }_{4}{X}_{i}+{\mu }_{i}.$$

Our coefficients of interest in these two equations are ${\gamma }_{3}$ and ${\theta }_{3}$ that indicate how the financial literacy score could change the effects of behavioral traits on the adoption and usage frequency of ePayment services $F{T}_{i}$ and $F{T}_{i}^{*}$, respectively. All variables are measured as previously described.

The coefficient estimates of the financial literacy variable may be biased due to reverse causality (i.e., using ePayment services may help adopters improve their financial literacy) and omitted variables, such as unobserved motivations and abilities, which affect both the adoption of ePayment services and financial literacy. To address these potential endogeneity problems, we use two IVs. First, following Fernandes et al. (2014) and Murendo and Mutsonziwa (2017), we use the mean financial literacy score at the prefectural level as the first instrument for individual financial literacy. Second, we use information from the question “Are you aware of the lowering of the age of adulthood?” In 2018, the Japanese government approved an amendment to the country’s civil code, which reduced the age of legal adulthood by two years to 18, effective April 1, 2022. This change is essential for two reasons. First, it significantly affected youths, their guardians, and those who cared for them. Second, it changed a law that had been in effect for more than 140 years. The rationale for using this instrument is that those aware of this change are more likely to be interested in accumulating socio-economic knowledge. As improving financial literacy is a form of human capital accumulation (Lusardi and Mitchell 2014), awareness of changes in the age of adulthood is expected to correlate positively with financial literacy. Simultaneously, this may not necessarily correlate with adopting and using fintech services. Although this variable has not been used in other studies, it aligns with those that instrument financial literacy by acquiring economic and financial knowledge (Hsiao and Tsai 2018).

### Variable construction

There are two independent variables in our analysis. We construct our dependent variables using information from these questions: “How often do you use e-money?” and “How often do you use [mobile] payment apps?” The answers to these two questions are “Almost every day,” “About once a week,” “About once a month,” “I hardly ever use,” and “I don't use.” The first dependent variable is related to the adoption of ePayment services. This binary variable takes a value of one if a person uses either e-money or mobile payment apps at least once a month and zero otherwise. The second dependent variable is the usage frequency of e-money or mobile payment apps. Using the same information, we construct the following four levels of intensity of use for each product: (i) daily, (ii) once a week, (iii) once a month, and (iv) no use.

We use a set of 25 questions from the survey to calculate a financial literacy index. This set consists of 18 questions on financial transactions; economic and financial knowledge; and knowledge of wealth-building, insurance, lending, and borrowing. In addition, there are seven questions on financial decision-making skills, such as household budget management, life planning skills, and outside expertise.^7^ The financial literacy score is calculated as the number of correct answers, ranging from 0 to 25. For ease of interpretation, we calculate the z-score of the financial literacy score.

We examine two types of behavioral traits: risk aversion and herd behavior. For risk aversion, we use information from the question “Suppose you invested 100,000 yen, there is an equal probability that you would either gain 20,000 yen or lose 10,000 yen. What would you do?” People are viewed as risk-averse if they answer “No, I would not invest.” For herd behavior, we use information from the question “How much do you agree or disagree that the statement ‘When there are several similar products, I tend to buy what is recommended as the highest-selling product rather than what I actually think is a good product’ applies to you personally?” People are considered to have herd behavior if they answer “Very much agree” or “Somewhat agree.” If they answer “Neutral,” “Somewhat disagree,” or “Disagree,” they are considered to not have herd behavior.

${X}_{i}$ is a vector of control variables that may influence the adoption of ePayment services. The first set of control variables is derived from the implications of the UTAUT2 Model. We control for individuals’ perception of the availability of stores that accept ePayment services (as a proxy for the facilitation condition), the time to process payment (as another proxy for the facilitation condition), the perception regarding managing private information and tools to control overpayment (as proxies for perceived risks), and the perception of cash usage (as a proxy for performance expectancy) based on their responses to the relevant questions in the survey. We also include individual characteristics such as age group, sex, level of general education (dummies), financial education, income (dummies), occupation, and frequency of reading financial and economic news.^8^

### Descriptive analysis

Table 1 presents the descriptive statistics of the sample. Owing to missing information, our analysis is based on 24,516 observations. The average financial literacy score is 14.1 (standard deviation: 6.9). Approximately 35.8% and 8.0% of the respondents in our sample use e-money and mobile payment apps, respectively, and 38.0% use at least one ePayment service. Regarding behavioral traits, 77.3% and 16.7% of the respondents are classified as risk-averse and exhibiting herd behavior, respectively. Surprisingly, nearly 25% of the respondents are fully satisfied with cash payments. Only 18.5% think that private information is not secure, and 10% state that tools to prevent the overuse of ePayment services are lacking. More than 23% of the respondents report that a limited number of stores accept ePayment services.

**Table 1:** Descriptive statistics *Source*: Authors’ calculation

	Mean	SD
Financial literacy score	14.15	6.87
e-money adopter	35.78	47.94
AppMoney adopter	7.97	27.09
ePayment adopter	37.96	48.53
Risk aversion	77.30	41.89
Herd behavior	16.72	37.32
Limited acceptance (of stores)	23.16	42.19
Long time for settlement	8.73	28.23
Private information not adequately secure	18.54	38.87
Lack of tools to prevent overuse	10.48	30.63
Fully satisfied with cash	24.49	43.01
Male	49.45	50.00
Age group		
Age < 30 (%)	15.03	35.74
Age ≥ 30 and < 40	16.04	36.69
Age ≥ 40 and < 50	19.10	39.31
Age ≥ 50 and < 60	16.12	36.77
Age ≥ 60 and < 70	19.24	39.42
Age ≥ 70	14.48	35.19
Education		
Primary/secondary/others	2.90	16.78
High school	32.41	46.81
Specialized college	11.24	31.59
Junior college/technical college	11.33	31.69
University	38.20	48.59
Graduate school	3.92	19.41
Income (Yen)		
No income	3.18	17.56
< 2.5 ml	15.68	36.36
≥ 2.5 ml and < 5 ml	28.32	45.06
≥ 5 ml and < 7.5 ml	17.32	37.84
≥ 7.5 ml and < 10 ml	9.84	29.79
≥ 10 ml and < 15 ml	5.38	22.56
≥ 15 ml	1.91	13.68
Don't know/don’t say	18.37	38.73

Table 2 presents a brief overview of the respondents who use e-money, mobile payment apps, and at least one ePayment service. On average, their financial literacy is higher than average. Risk-averse people are less likely to use ePayment services than non-risk-averse people. However, the difference in the adoption rate is not different between those with and without herd behavior. It is interesting to note that the proportion of those who claim that the number of stores accepting ePayment services is limited, it takes more time for payment settlement, and tools to prevent overuse and protect private information are lacking among ePayment users is slightly higher than non ePayment users. In addition, the adoption rates among men, younger persons, more educated persons, and those with higher incomes are higher than those among women, older persons, less educated persons, and those with lower incomes.

**Table 2:** Statistics of adopters of ePayment services *Source*: Authors’ calculation

	E-money adoption		Mobile payment app adoption		Adoption of at least one ePayment service	
	Mean	SD	Mean	SD	Mean	SD
Financial literacy	15.69	6.35	15.20	6.58	15.66	6.37
With risk aversion (%)	33.43	47.17	6.02	23.79	35.20	47.76
Without risk aversion (%)	43.81	49.62	14.61	35.32	47.34	49.93
With herd behavior (%)	36.69	48.20	11.79	32.25	39.78	48.95
Without herd behavior (%)	35.60	47.88	7.20	25.86	37.59	48.44
Limited acceptance (of stores) (%)	38.97	48.77	7.96	27.07	41.58	49.29
Long time to settle (%)	34.98	47.70	7.24	25.92	37.60	48.45
Private information not adequately secure (%)	36.32	48.10	2.38	15.23	37.15	48.33
Lack of tools to prevent overuse (%)	34.55	47.56	5.64	23.08	36.65	48.20
Fully satisfied with cash (%)	24.21	42.84	3.88	19.31	25.18	43.41
Female (%)	34.93	47.68	5.05	21.89	36.51	48.15
Male (%)	36.65	48.19	10.96	31.24	39.44	48.87
Age < 30 (%)	31.93	46.63	11.81	32.28	35.66	47.91
Age ≥ 30 and < 40 (%)	36.94	48.27	12.30	32.84	40.43	49.08
Age ≥ 40 and < 50 (%)	39.25	48.84	9.80	29.74	41.73	49.32
Age ≥ 50 and < 60 (%)	41.62	49.30	7.64	26.57	43.26	49.55
Age ≥ 60 and < 70 (%)	36.29	48.09	4.30	20.29	37.52	48.42
Age ≥ 70 (%)	26.75	44.27	2.02	14.06	27.30	44.56
Primary/secondary/others (%)	23.59	42.48	5.93	23.64	25.10	43.39
High school (%)	29.69	45.69	6.28	24.26	31.83	46.58
Specialized college (%)	34.08	47.41	7.15	25.77	36.43	48.13
Junior college/technical college (%)	36.19	48.06	5.23	22.26	37.71	48.48
University graduate (%)	40.97	49.18	10.01	30.02	43.25	49.54
Graduate school (%)	48.37	50.00	13.88	34.59	51.63	50.00
No income (%)	21.73	41.27	4.27	20.23	22.99	42.10
< 2.5 ml (%)	32.05	46.67	6.58	24.80	34.01	47.38
≥ 2.5 ml and < 5 ml (%)	34.67	47.60	7.22	25.88	37.09	48.31
≥ 5 ml and < 7.5 ml (%)	40.24	49.04	9.56	29.41	42.71	49.47
≥ 7.5 ml and < 10 ml (%)	44.43	49.70	11.30	31.67	47.40	49.94
≥ 10 ml and < 15 ml (%)	49.37	50.01	13.75	34.46	51.75	49.99
≥ 15 ml (%)	52.41	49.99	16.77	37.40	54.72	49.83
Don't know/don’t say (%)	28.59	45.19	5.07	21.95	29.94	45.80

## Estimation results

### Benchmark estimations

#### Adoption of ePayment services

Table 3 presents our benchmark results after estimating Eq. (1) using the ordinary least squares (OLS) method. The dependent variable in columns 1, 2 and 3 show whether an individual uses e-money, mobile payment apps, and at least one ePayment service, respectively.^9^^,^ 10 Our empirical results show that financial literacy positively correlates with adopting e-money and mobile payment apps and using at least one ePayment service. Specifically, a one-standard-deviation increase in financial literacy is associated with a 5.3%, 1.2%, and 5.9% higher likelihood of using e-money, mobile payment apps, and at least one ePayment service, respectively. This result supports H1.

**Table 3:** Financial literacy, behavioral traits, and ePayment adoption: OLS estimation *Source*: Authors’ estimation

	Using e-money	Using mobile payment apps	Using either e-money or mobile payment apps
	(1)	(2)	(3)
Financial literacy	0.053***	0.012***	0.059***
	[0.004]	[0.002]	[0.004]
Risk aversion	− 0.029***	− 0.034***	− 0.037***
	[0.008]	[0.005]	[0.008]
Herd behavior	0.003	0.017***	0.008
	[0.008]	[0.005]	[0.008]
Limited acceptance (at stores)	− 0.008	− 0.010**	− 0.008
	[0.008]	[0.004]	[0.008]
Long time for settlement	− 0.017	− 0.013**	− 0.016
	[0.011]	[0.006]	[0.011]
Private information not adequately secure	− 0.041***	− 0.061***	− 0.058***
	[0.008]	[0.004]	[0.008]
Lack of tools to prevent overuse	− 0.037***	− 0.019***	− 0.039***
	[0.010]	[0.005]	[0.010]
Fully satisfied with cash	− 0.078***	− 0.042***	− 0.091***
	[0.008]	[0.004]	[0.008]
Male	− 0.046***	0.020***	− 0.039***
	[0.008]	[0.004]	[0.008]
Age group (reference group: ≤ 30 years old)			
30–40 years old	0.041***	0.001	0.033***
	[0.011]	[0.008]	[0.012]
40–50 years old	0.050***	− 0.029***	0.032***
	[0.011]	[0.007]	[0.012]
50–60 years old	0.070***	− 0.048***	0.043***
	[0.012]	[0.007]	[0.012]
60–70 years old	0.041***	− 0.063***	0.012
	[0.012]	[0.007]	[0.012]
≥ 70 years old	− 0.026**	− 0.076***	− 0.060***
	[0.013]	[0.007]	[0.013]
Education (reference group: primary and junior high school)			
High school	− 0.006	− 0.000	− 0.003
	[0.016]	[0.008]	[0.016]
Specialized college	0.012	− 0.008	0.011
	[0.018]	[0.009]	[0.018]
Junior college/technical college	0.022	− 0.002	0.022
	[0.018]	[0.009]	[0.018]
University	0.043***	− 0.002	0.039**
	[0.016]	[0.009]	[0.017]
Graduate school	0.081***	0.002	0.080***
	[0.022]	[0.013]	[0.023]
Income (JPY) (reference group: no income)			
< 2.5 ml	0.067***	0.020**	0.074***
	[0.016]	[0.008]	[0.017]
≥ 2.5 ml and < 5 ml	0.073***	0.021**	0.081***
	[0.017]	[0.008]	[0.017]
≥ 5 ml and < 7.5 ml	0.088***	0.027***	0.095***
	[0.018]	[0.009]	[0.018]
≥ 7.5 ml and < 10 ml	0.097***	0.037***	0.109***
	[0.019]	[0.010]	[0.019]
≥ 10 ml and < 15 ml	0.116***	0.052***	0.124***
	[0.021]	[0.012]	[0.021]
≥ 15 ml	0.134***	0.065***	0.144***
	[0.028]	[0.017]	[0.028]
Don't report income	0.048***	0.021***	0.053***
	[0.016]	[0.008]	[0.017]
Occupation dummies	Yes	Yes	Yes
Frequency of news acquired dummies	Yes	Yes	Yes
Prefectural dummies	Yes	Yes	Yes
Intercept	0.271***	0.098***	0.309***
	[0.029]	[0.015]	[0.029]
N	24,516	24,516	24,516

*, **, *** Indicate significance at the 10%, 5%, and 1% levels, respectively. Standard errors are in brackets

The estimation results also indicate that risk aversion is negatively associated with the likelihood of using e-money, mobile payment apps, and at least one ePayment service. However, the coefficient of the herd behavior variable is positively correlated with the likelihood of using mobile payment apps but not with that of using e-money and at least one ePayment service.

Our estimation results also show that a limited number of stores accepting ePayment services and a long time to process payments are negatively associated with adopting mobile payment apps. Meanwhile, issues related to risks and risk management, such as inappropriate securing of private information or a lack of tools to prevent overuse (of these payments), are negatively correlated with the likelihood of adopting both e-money and mobile payment apps. Finally, those fully satisfied with cash payments are less likely to adopt ePayment services.

Regarding the other control variables, on average, men are less likely than women to adopt payment services. Individuals’ ages are also correlated with the likelihood of adopting ePayment services. More specifically, those aged 30–70 years are more likely to use e-money than those under 30, while the latter are more likely to adopt mobile payment apps. People over 70 are less likely to adopt e-money and mobile payment apps than those under 30. The results also suggest that individuals with at least an undergraduate degree are more likely to adopt e-money. However, the educational level does not correlate with the likelihood of adopting mobile payment apps. Those with higher incomes are likely to use e-money or mobile payment apps.

To deal with potential endogeneity problems in financial literacy scores, we use the IV approach. The estimated results are presented in Table 4. Column 1 presents the first-stage estimation results, and columns 2–4 show the estimation results using the IVs. The first-stage results show that all IVs are related to financial literacy, with statistical significance at the 1% and 5% levels. In all equations, the Anderson Wald and Cragg–Donald Wald test statistics indicate that our IVs do not suffer from under-identification or weak instrument problems, respectively. In addition, the Sargan test indicates that our IVs satisfy the over-identification conditions.

**Table 4:** Financial literacy, behavioral traits and ePayment adoption: IV estimation *Source*: Authors’ estimation

	First stage estimation	Using e-money	Using mobile payment apps	Using either e-money or mobile payment apps
		(1)	(2)	(3)
Average financial literacy	0.097***			
	[0.008]			
Awareness of change in adulthood age	0.618***			
	[0.014]			
Financial literacy		0.108***	0.031***	0.117***
		[0.012]	[0.006]	[0.012]
Risk aversion	− 0.234***	− 0.015*	− 0.029***	− 0.022***
	[0.012]	[0.008]	[0.005]	[0.008]
Herd behavior	− 0.183***	0.014*	0.021***	0.020**
	[0.014]	[0.008]	[0.005]	[0.009]
Limited acceptance (at stores)	0.132***	− 0.017**	− 0.013***	− 0.017**
	[0.012]	[0.008]	[0.004]	[0.008]
Long time for settlement	− 0.171***	− 0.007	− 0.009	− 0.006
	[0.018]	[0.011]	[0.006]	[0.011]
Private information not adequately secure	0.228***	− 0.056***	− 0.065***	− 0.073***
	[0.012]	[0.009]	[0.004]	[0.009]
Lack of tools to prevent overuse	0.069***	− 0.042***	− 0.021***	− 0.045***
	[0.015]	[0.010]	[0.005]	[0.010]
Fully satisfied with cash	− 0.219***	− 0.064***	− 0.037***	− 0.076***
	[0.015]	[0.009]	[0.005]	[0.009]
Male	0.062***	− 0.048***	0.019***	− 0.042***
	[0.013]	[0.008]	[0.004]	[0.008]
Age group (reference group: ≤ 30 years old)				
30–40 years old	− 0.034	0.032***	− 0.002	0.024**
	[0.025]	[0.012]	[0.008]	[0.012]
40–50 years old	− 0.042	0.035***	− 0.035***	0.016
	[0.031]	[0.012]	[0.007]	[0.012]
50–60 years old	− 0.052	0.048***	− 0.056***	0.019
	[0.040]	[0.013]	[0.008]	[0.013]
60–70 years old	− 0.058	0.012	− 0.073***	− 0.018
	[0.046]	[0.013]	[0.008]	[0.013]
≥ 70 years old	− 0.065	− 0.056***	− 0.086***	− 0.091***
	[0.048]	[0.014]	[0.008]	[0.014]
Education (reference group: primary and junior high school)				
High school	0.229***	− 0.021	− 0.005	− 0.019
	[0.029]	[0.016]	[0.008]	[0.017]
Specialized college	0.214***	− 0.002	− 0.013	− 0.004
	[0.031]	[0.018]	[0.009]	[0.018]
Junior college/technical college	0.305***	0.003	− 0.009	0.001
	[0.032]	[0.018]	[0.009]	[0.019]
University	0.487***	0.012	− 0.013	0.006
	[0.029]	[0.017]	[0.009]	[0.018]
Graduate school	0.664***	0.040*	− 0.013	0.036
	[0.038]	[0.024]	[0.014]	[0.024]
Income (JPY) (reference group: no income)				
< 2.5 ml	0.188***	0.054***	0.016*	0.059***
	[0.032]	[0.017]	[0.008]	[0.017]
≥ 2.5 ml and < 5 ml	0.280***	0.053***	0.014	0.060***
	[0.032]	[0.017]	[0.009]	[0.018]
≥ 5 ml and < 7.5 ml	0.372***	0.063***	0.018*	0.068***
	[0.034]	[0.019]	[0.009]	[0.019]
≥ 7.5 ml and < 10 ml	0.425***	0.068***	0.027**	0.078***
	[0.035]	[0.020]	[0.010]	[0.020]
≥ 10 ml and < 15 ml	0.414***	0.088***	0.042***	0.094***
	[0.038]	[0.022]	[0.012]	[0.022]
≥ 15 ml	0.354***	0.109***	0.057***	0.118***
	[0.050]	[0.028]	[0.017]	[0.028]
Don't report income	0.000	0.045***	0.020**	0.050***
	[0.032]	[0.016]	[0.008]	[0.017]
Occupation dummies	Yes	Yes	Yes	Yes
Frequency of news acquired dummies	Yes	Yes	Yes	Yes
Prefectural dummies	Yes	Yes	Yes	Yes
Intercept	− 2.128***	0.312***	0.113***	0.352***
	[0.094]	[0.030]	[0.016]	[0.031]
Anderson canon. corr. LM statistic		1653.865	1653.865	1653.865
Cragg–Donald Wald *F* statistic		1258.159	1258.159	1258.159
Sargan statistics (*p*-value)		0.3196	0.3804	0.7757
N	24,516	24,516	24,516	24,516

*, ** and *** indicate significance at the 10%, 5%, and 1% levels, respectively. Standard errors are in brackets

The estimates of the effect of financial literacy are larger than those obtained from the OLS estimation (Table 3). Specifically, a one-standard-deviation increase in financial literacy is associated with a 10.8%, 3.1% and 11.7% higher likelihood of using e-money, mobile payment apps, and at least one ePayment service, respectively. This result is consistent with most studies that use IVs for financial literacy, such as Bucher-Koenen and Lusardi (2011), Agnew et al. (2013), and Morgan and Long (2020). According to Lusardi and Mitchell (2014), this downward bias may be explained by measurement errors when calculating financial literacy. The OLS downward bias may also occur because those affected by the instruments responded better (van Rooij et al. 2011).

The estimation results obtained from the IV approach also slightly change the relationship between the behavioral trait variables and adopting ePayment services. While risk aversion is still negatively correlated with adopting ePayment services, herd behavior is positively correlated with adopting both e-money and mobile payment apps. However, this result is partly because these two variables correlate with financial literacy, as shown in column 1.

#### Usage frequency of ePayment services

We further explore the effects of financial literacy and behavioral traits on the usage frequency of ePayment services. Table 5 presents the marginal effects of financial literacy, behavioral traits, and other factors on the intensity of using e-money (columns 1–4) and mobile payment apps (columns 5–8). The results are obtained by estimating the ordered probit regression presented in Eq. (2). We adopt Wooldridge’s (2010) approach to estimate ordered probit models with endogenous variables. The two IVs described above are also used. The first-stage regression results are similar to those in Table 4 (Column 1).

**Table 5:** Financial literacy, behavioral traits and frequency of ePayment usage: ordered probit estimation *Source*: Authors’ estimation

	E-money				Mobile payment apps			
	Almost every day	Once a week	Once a month	Never	Almost every day	Once a week	Once a month	Never
	(1)	(2)	(3)	(4)	(5)	(6)	(7)	(8)
Financial literacy	0.028***	0.031***	0.004***	− 0.064***	0.003***	0.006***	0.007***	− 0.015***
	[0.002]	[0.002]	[0.000]	[0.003]	[0.001]	[0.001]	[0.001]	[0.003]
Risk aversion	− 0.013***	− 0.014***	− 0.002***	0.029***	− 0.008***	− 0.015***	− 0.018***	0.041***
	[0.003]	[0.003]	[0.000]	[0.007]	[0.001]	[0.002]	[0.002]	[0.005]
Herd behavior	0.003	0.003	0.000	− 0.007	0.004***	0.007***	0.009***	− 0.019***
	[0.003]	[0.004]	[0.000]	[0.007]	[0.001]	[0.002]	[0.003]	[0.006]
Limited acceptance (at stores)	− 0.006**	− 0.007**	− 0.001**	0.015**	− 0.001	− 0.002	− 0.003	0.006
	[0.003]	[0.003]	[0.000]	[0.006]	[0.001]	[0.002]	[0.002]	[0.005]
Long time for settlement	− 0.008*	− 0.009*	− 0.001*	0.018*	− 0.003**	− 0.007**	− 0.008**	0.018**
	[0.004]	[0.005]	[0.001]	[0.010]	[0.001]	[0.003]	[0.004]	[0.008]
Private information not adequately secure	− 0.019***	− 0.021***	− 0.003***	0.044***	− 0.021***	− 0.042***	− 0.051***	0.114***
	[0.003]	[0.003]	[0.000]	[0.007]	[0.002]	[0.003]	[0.003]	[0.007]
Lack of tools to prevent overuse	− 0.011***	− 0.013***	− 0.002***	0.026***	− 0.001	− 0.002	− 0.002	0.004
	[0.004]	[0.004]	[0.001]	[0.009]	[0.001]	[0.003]	[0.003]	[0.007]
Fully satisfied with cash	− 0.047***	− 0.052***	− 0.007***	0.106***	− 0.011***	− 0.023***	− 0.028***	0.062***
	[0.004]	[0.004]	[0.001]	[0.008]	[0.001]	[0.002]	[0.003]	[0.006]
Male	− 0.020***	− 0.022***	− 0.003***	0.044***	0.001	0.003	0.003	− 0.007
	[0.003]	[0.003]	[0.000]	[0.007]	[0.001]	[0.002]	[0.002]	[0.005]
Age group (reference group: ≤ 30)								
30–40 years old	0.012**	0.014**	0.002**	− 0.027**	− 0.003	− 0.006	− 0.006	0.015
	[0.005]	[0.006]	[0.001]	[0.011]	[0.002]	[0.004]	[0.004]	[0.010]
40–50 years old	0.017***	0.019***	0.003***	− 0.038***	− 0.015***	− 0.027***	− 0.029***	0.071***
	[0.005]	[0.005]	[0.001]	[0.011]	[0.002]	[0.004]	[0.004]	[0.010]
50–60 years old	0.026***	0.028***	0.003***	− 0.056***	− 0.021***	− 0.041***	− 0.047***	0.110***
	[0.005]	[0.006]	[0.001]	[0.011]	[0.002]	[0.004]	[0.004]	[0.010]
60–70 years old	0.011**	0.012**	0.002**	− 0.025**	− 0.027***	− 0.056***	− 0.068***	0.151***
	[0.005]	[0.006]	[0.001]	[0.011]	[0.002]	[0.004]	[0.004]	[0.010]
≥ 70 years old	− 0.016***	− 0.021***	− 0.005***	0.041***	− 0.032***	− 0.072***	− 0.097***	0.200***
	[0.005]	[0.006]	[0.001]	[0.013]	[0.002]	[0.004]	[0.005]	[0.009]
Education (reference group: primary and junior high school)								
High school	0.009	0.012	0.003	− 0.024	− 0.001	− 0.002	− 0.003	0.006
	[0.007]	[0.010]	[0.002]	[0.019]	[0.003]	[0.005]	[0.006]	[0.014]
Specialized college	0.016**	0.020**	0.004*	− 0.040**	− 0.004	− 0.008	− 0.010	0.022
	[0.007]	[0.010]	[0.002]	[0.020]	[0.003]	[0.006]	[0.006]	[0.015]
Junior college/technical college	0.021***	0.026***	0.005**	− 0.053***	− 0.004	− 0.008	− 0.009	0.020
	[0.008]	[0.010]	[0.002]	[0.020]	[0.003]	[0.006]	[0.007]	[0.015]
University	0.030***	0.036***	0.006***	− 0.072***	− 0.004	− 0.008	− 0.009	0.021
	[0.007]	[0.010]	[0.002]	[0.019]	[0.003]	[0.005]	[0.006]	[0.014]
Graduate school	0.048***	0.053***	0.006***	− 0.108***	− 0.004	− 0.008	− 0.010	0.023
	[0.010]	[0.011]	[0.002]	[0.022]	[0.003]	[0.006]	[0.008]	[0.017]
Income (JPY) (reference group: no income)								
< 2.5 ml	0.027***	0.036***	0.008***	− 0.071***	0.004**	0.009**	0.012**	− 0.025**
	[0.006]	[0.009]	[0.003]	[0.018]	[0.002]	[0.004]	[0.006]	[0.012]
≥ 2.5 ml and < 5 ml	0.030***	0.038***	0.008***	− 0.076***	0.006***	0.014***	0.018***	− 0.037***
	[0.006]	[0.009]	[0.003]	[0.018]	[0.002]	[0.005]	[0.006]	[0.013]
≥ 5 ml and < 7.5 ml	0.034***	0.044***	0.009***	− 0.087***	0.007***	0.015***	0.019***	− 0.041***
	[0.007]	[0.009]	[0.003]	[0.019]	[0.002]	[0.005]	[0.006]	[0.013]
≥ 7.5 ml and < 10 ml	0.040***	0.049***	0.009***	− 0.098***	0.009***	0.020***	0.025***	− 0.054***
	[0.007]	[0.010]	[0.003]	[0.019]	[0.002]	[0.005]	[0.007]	[0.014]
≥ 10 ml and < 15 ml	0.042***	0.051***	0.010***	− 0.103***	0.013***	0.026***	0.033***	− 0.072***
	[0.008]	[0.010]	[0.003]	[0.021]	[0.003]	[0.006]	[0.007]	[0.016]
≥ 15 ml	0.053***	0.062***	0.010***	− 0.125***	0.019***	0.036***	0.043***	− 0.098***
	[0.011]	[0.013]	[0.003]	[0.025]	[0.005]	[0.008]	[0.009]	[0.022]
Don't report income	0.019***	0.026***	0.006**	− 0.050***	0.004**	0.010**	0.013**	− 0.028**
	[0.006]	[0.009]	[0.003]	[0.018]	[0.002]	[0.005]	[0.006]	[0.013]
Occupation dummies	Yes	Yes	Yes	Yes	Yes	Yes	Yes	Yes
Frequency of news acquired dummies	Yes	Yes	Yes	Yes	Yes	Yes	Yes	Yes
Prefectural dummies	Yes	Yes	Yes	Yes	Yes	Yes	Yes	Yes
N	24,516	24,516	24,516	24,516	24,516	24,516	24,516	24,516

*, **, *** Indicate significance at the 10%, 5%, and 1% levels, respectively. Standard errors are in brackets

Our estimation results demonstrate that if an individual’s financial literacy increases by one standard deviation, it is associated with a 2.8%, 3.1%, and 0.4% higher likelihood of using e-money daily, once a week, and once a month, respectively. Similarly, the likelihood of using mobile payment apps daily, once a week, and once a month increases by 0.3, 0.6, and 0.7 percentage points, respectively. As expected, the likelihood of not using e-money and mobile payment apps is reduced by 6.4 and 1.5 percentage points, respectively.

The estimation results also indicate that risk aversion reduces the likelihood of e-money use almost daily and once a week by approximately 1.3 and 1.4 percentage points, respectively, while increasing the likelihood of not using it by 2.9 percentage points. A similar pattern is observed for the likelihood of adopting mobile payment apps. Herd behavior, however, is not related to the adoption of e-money but is strongly correlated with the frequent usage of mobile payment apps.

We also find that a limited number of stores accepting ePayment services, a long time to process payments, insufficient protection of private information, a lack of tools to prevent overuse, and being fully satisfied with cash payments are positively correlated with the frequent use of e-money. However, a limited number of stores accepting ePayment services and a lack of tools to prevent overuse do not correlate with the usage frequency of mobile payment apps.

Concerning age, for using ePayment services daily, once a week, once a month, or never, middle-aged individuals (aged 40–69 years) are more likely to use e-money than younger ones but are less likely to use mobile payment apps. Meanwhile, for all three outcomes, younger people (under 30) tend to use mobile payment apps more than older people. People in all age groups use mobile payment apps once a week or once a month rather than daily.

### Heterogeneous effects of financial literacy by behavioral traits

Table 6 presents our estimation results using Eq. (3). The effects of financial literacy on those with and without risk aversion and those with and without herd behavior are presented in Panels A and B, respectively. We use the IV approach to estimate all the specifications. The first-stage estimation results for Panels A and B are presented in Appendix 1. In all specifications, the Anderson Wald and Cragg–Donald Wald test statistics indicate that our IVs do not suffer from under-identification or weak instrument problems. In addition, the Sargan test indicates that our IVs satisfy the over-identification conditions.

**Table 6:** Heterogeneous effects of financial literacy on ePayment adoption by behavioral traits (IV estimations) *Source*: Authors’ estimation

	Using e-money	Using mobile payment apps	Using either e-money or mobile payment apps
	(1)	(2)	(3)
Panel A:			
Financial literacy	0.101***	0.039**	0.084***
	[0.030]	[0.019]	[0.031]
Financial literacy * Risk aversion	0.004	0.041***	0.019
	[0.016]	[0.010]	[0.016]
Risk aversion	− 0.017	− 0.056***	− 0.034***
	[0.013]	[0.009]	[0.013]
Herd behavior	0.014	0.017***	0.019**
	[0.009]	[0.005]	[0.009]
Other covariates			
Anderson canon. corr. LM statistic	440.445	440.445	440.445
Cragg–Donald Wald *F* statistic	502.005	502.005	502.005
Sargan statistics (*p*-value)	0.3259	0.5525	0.8312
Panel B:			
Financial literacy	0.118***	0.035***	0.127***
	[0.013]	[0.007]	[0.013]
Financial literacy * Herd behavior	− 0.054*	− 0.022	− 0.057*
	[0.030]	[0.018]	[0.031]
Herd behavior	0.004	0.017***	0.010
	[0.010]	[0.005]	[0.010]
Risk aversion	− 0.015*	− 0.029***	− 0.022***
	[0.008]	[0.005]	[0.008]
Other covariates			
Anderson canon. corr. LM statistic	433.22	433.22	433.22
Cragg–Donald Wald *F* statistic	606.369	606.369	606.369
Sargan statistics (*p*-value)	0.3124	0.3744	0.7636

*, **, ***Indicate significance at the 10%, 5%, and 1% levels, respectively. Standard errors are in brackets. Our regressions also control for occupation, frequency of acquiring news, and prefectural dummies. The first stage estimation results are presented in Appendix 1

The results in Panel A show that financial literacy is still strongly and positively correlated with adopting e-money, mobile payment apps, and at least one ePayment service. Among those with risk aversion, higher financial literacy is associated with a higher likelihood of adopting mobile payment apps but not e-money. However, the results shown in Panel B provide a slightly different perspective. While we still find that financial literacy is important in determining one’s decision to adopt any ePayment service, among those with herd behavior, higher financial literacy is negatively associated with using e-money but not mobile payment apps.

Table 7 reports our results on the usage frequency of ePayment services. We also use Wooldridge’s (2010) approach to address the endogeneity of financial literacy in these estimates. The first-stage results are similar to those in Table 6 and presented in Appendix 1. Panels A and B present the heterogeneous effects of financial literacy on the frequent use of ePayment services based on two behavioral traits: risk aversion and herd behavior. The results in Panel A show that the effect of financial literacy on the usage frequency of e-money is greater for risk-averse individuals. However, a higher level of financial literacy is not correlated with a higher frequency of mobile payment app use among risk-averse individuals. Meanwhile, risk aversion is negatively correlated with more frequent usage of both e-money and mobile payment apps. The results also suggest that financial literacy reduces the negative biases caused by risk aversion in the frequent use of e-money but not in the frequent use of mobile payment apps. The results in Panel B show that the effect of financial literacy does not differ between those with and without herd behavior in terms of the usage frequency of both ePayment services. However, those with herd behavior tend to use ePayment services more frequently.

**Table 7:** Heterogeneous effects of financial literacy on ePayment usage by behavioral traits (ordered probit estimation) *Source*: Authors’ estimation

	E-money				Mobile payment apps			
	Almost every day	Once a week	Once a month	Never	Almost every day	Once a week	Once a month	Never
	(1)	(2)	(3)	(4)	(5)	(6)	(7)	(8)
Panel A:								
Financial literacy	0.051***	0.056***	0.007***	− 0.115***	0.010***	0.019***	0.024***	− 0.053***
	[0.007]	[0.007]	[0.001]	[0.015]	[0.002]	[0.004]	[0.005]	[0.011]
Financial literacy * Risk aversion	0.011**	0.012**	0.002**	− 0.024**	− 0.001	− 0.003	− 0.004	0.008
	[0.005]	[0.006]	[0.001]	[0.012]	[0.002]	[0.003]	[0.004]	[0.009]
Risk aversion	− 0.008**	− 0.009**	− 0.001**	0.019**	− 0.006***	− 0.011***	− 0.014***	0.031***
	[0.004]	[0.004]	[0.001]	[0.008]	[0.001]	[0.002]	[0.003]	[0.006]
Herd behavior	0.009***	0.010***	0.001***	− 0.021***	0.005***	0.009***	0.011***	− 0.025***
	[0.003]	[0.004]	[0.000]	[0.008]	[0.001]	[0.002]	[0.003]	[0.006]
Other covariates	Yes	Yes	Yes	Yes	Yes	Yes	Yes	Yes
Panel B:								
Financial literacy	0.062***	0.068***	0.009***	− 0.138***	0.008***	0.017***	0.021***	− 0.046***
	[0.005]	[0.006]	[0.001]	[0.012]	[0.002]	[0.003]	[0.004]	[0.009]
Financial literacy * Herd behavior	− 0.009	− 0.009	− 0.001	0.019	0.000	0.001	0.001	− 0.002
	[0.005]	[0.006]	[0.001]	[0.012]	[0.002]	[0.003]	[0.004]	[0.009]
Herd behavior	0.008**	0.009**	0.001**	− 0.019**	0.005***	0.009***	0.011***	− 0.025***
	[0.003]	[0.004]	[0.000]	[0.008]	[0.001]	[0.002]	[0.003]	[0.006]
Risk aversion	− 0.013***	− 0.014***	− 0.002***	0.029***	− 0.006***	− 0.012***	− 0.015***	0.033***
	[0.003]	[0.003]	[0.000]	[0.007]	[0.001]	[0.002]	[0.003]	[0.006]
Other covariates	Yes	Yes	Yes	Yes	Yes	Yes	Yes	Yes

*, **, *** Indicate significance at the 10%, 5%, and 1% levels, respectively. Standard errors are in brackets. Our regressions also control for occupation, frequency of acquiring news, and prefectural dummies

## Discussion and concluding remarks

Although rapid developments in fintech are expected to improve financial inclusion and well-being, these services entail numerous traditional and new Internet-related risks (Li et al. 2021; Morgan et al. 2019), which require consumers to have the adequate financial literacy to make decisions about the adoption and use of fintech services. Using a large dataset collected in a survey by the Bank of Japan covering 25,000 individuals aged 18–79 years, this study investigated the effect of financial literacy on the extensive and intensive usages of fintech ePayment services. We also examined the heterogeneous effects of financial literacy on behavioral traits, that is, risk aversion and herd behavior.

First, financial literacy positively affects the adoption and usage of e-money, mobile payment apps, and at least one ePayment service. Specifically, a one-standard-deviation increase in financial literacy increases the likelihood of using e-money, mobile payment apps, and at least one ePayment service by 10.8, 3.1 and 11.7 percentage points, respectively. A one-standard-deviation increase in financial literacy is also associated with an increase of 2.8, 3.1 and 0.4 percentage points in the likelihood of using e-money daily, once a week, and once a month, respectively. The corresponding figure for mobile payment apps are 0.3, 0.6, and 0.7. The results are consistent, regardless of the estimation method and specifications. These results confirm H1. These results are consistent with those of previous studies (Hilgert et al. 2003; Christelis et al. 2010; van Rooij et al. 2011; Morgan and Long 2019; Foster and Johansyah 2021; Morgan and Long 2018). However, our findings differ from Li et al.’s (2020) and Chen and Xiang’s (2021) findings. They argue that people with better financial knowledge might recognize the risks involved and be less likely to adopt ePayment services. While we do not have a clear explanation for this difference, we controlled for risk behavior-related variables in our estimation. Thus, these two variables capture the negative relationship between financial knowledge and ePayment adoption.

Second, we find that the effect of financial literacy on the adoption and intensity of ePayment service usage differs for people with different behavioral traits. More specifically, we find evidence to confirm H2c, that is, with regard to e-money, financial literacy affects the usage frequency of individuals with and without risk aversion differently. Nevertheless, the evidence does not support H2a, which states that the effect of financial literacy on the adoption of e-money differs between individuals with and without risk aversion, and it could not be confirmed. Meanwhile, the effect of financial literacy on the adoption of mobile payment apps was greater among risk-averse individuals than among those without risk aversion. This result confirms H2b. However, our results indicate that a higher level of financial literacy does not correlate with more frequent use of mobile payment apps among risk-averse individuals. Thus, H2d could not be confirmed.

The difference in the results for the two types of payment services may be due to the differences in penetration rates. While e-money has been introduced since the early 2000s in many forms, including metro and bus cards, with more than 50% of the Japanese population owning at least one such instrument, mobile payment apps have recently been introduced (Bank of Japan 2022). Therefore, many people trust e-money but are still doubtful about mobile payment apps. Since adoption does not ensure frequent use of a product that has been implemented since a long time, such as e-money, the effect of financial literacy may differ for those with risk aversion. However, financial literacy may have a negligible effect on newer products, such as mobile payment apps. Our results further shed light on the mitigating role of financial literacy in reducing risk-aversion bias. As Jung (2015) argues, higher education may diminish risk aversion by providing people with a better understanding of risk management.

Third, the effect of financial literacy on e-money adoption does not differ between individuals with and without herd behavior. However, the effect of financial literacy on the adoption of mobile payment apps is lower for those with herd behavior. Thus, we could not confirm H3a, but we could confirm H3b. The different results for e-money and mobile payment apps may be due to the penetration rates of these two services. Individuals with herd behavior may adopt an older service with a high penetration rate, regardless of their financial literacy. Conversely, for a newer product with a low penetration rate, a higher level of financial literacy may encourage an individual with herd behavior to deviate from the herd and adopt the new financial product. We also find that financial literacy has no different effects on the usage frequency of both ePayment services; thus, H3c and H3d are not confirmed.

Fourth, behavioral biases affect the adoption of ePayment services. This result is consistent with other studies that show that risk attitudes and herd behavior affect individuals’ financial decisions (Badarinza et al. 2016; Barberis et al. 2006; Lin et al. 2013; Hong et al. 2004). However, the effects of behavioral biases differ by the type of behavioral trait and type of ePayment service. Risk aversion is negatively correlated with adopting e-money, mobile payment apps, and at least one ePayment service. Risk aversion hinders the frequent use of both e-money and mobile payment apps. This result is similar to the effect of risk aversion on risky financial behavior (Lin et al. 2013; Hong et al. 2004). This result also reflects that these ePayment services are still in the early stages, and thus, many people still perceive that the potential risks are high, discouraging them from using these services. We also find that herd behavior is positively correlated with adopting e-money, mobile payment apps, and at least one ePayment service. This result is consistent with previous studies that show the importance of peers in determining portfolio choices and stock market participants (Hong et al. 2004; van Rooij et al. 2011).

Fifth, the infrastructure for ePayment adoption and usage plays an important role. Our results show that having a limited number of stores that accept both e-money and mobile payment apps hinders the adoption and frequent use of these services. In addition, the lack of information protection and risk-management tools discourages the adoption of ePayment services. Our results are consistent with those of other studies that have used the UTAUT2 Model as an analytical framework, such as Shahzad et al. (2018), Abbasi et al. (2022) and Picoto and Pinto (2021).

### Theoretical, practical, and policy implications

The empirical evidence from this study provides important practical and theoretical contributions. The findings of this study also provide some theoretical implications for extending current theories on fintech services. First, they allow us to understand the factors determining the adoption of fintech products. Previous literature has shown that perceived usefulness, perceived costs (derived from the TAM), performance expectancy, perceived risk, perceived trust, and facilitation conditions (derived from the UTAUT2 Model) are important. Our results further confirm that financial literacy strongly affects the adoption and usage of fintech products regardless of the position of the services in their life cycles. The potential channels through which financial literacy affects the adoption of fintech products are user perceptions of the usefulness and cost of adopting fintech services. Second, our results add to the current theories on the effects of behavioral biases on financial decisions in general and the adoption of fintech services in particular. Existing theories do not consider the behavioral factors that affect the adoption of fintech services, partly because behavioral factors, such as risk aversion, may be related to the perceived risks or costs of fintech adoption or perceived trust or usefulness, such as herd behavior. Third, our results also provide some evidence to extend the current literature on the heterogeneity of the effects of financial literacy according to individuals’ behavioral traits. Simultaneously, our results also provide some evidence of the moderating role of financial literacy on the effect of behavioral traits on the financial decision-making process. Fourth, the empirical findings provide further evidence that current theories on technological adoption can be successfully extended and applied to a new setting and context, namely, fintech adoption and usage in Japan.

This study has significant implications for fintech developers and financial institutions seeking to provide services via the Internet. These findings highlight the importance of understanding the factors that shape the fintech service infrastructure. First, users’ financial literacy must be strengthened to improve the perceived costs, risks, and usefulness of fintech services. Financial literacy can also help users recognize the ease of using such services. Second, designing effective campaigns requires consideration of behavioral biases, including risk aversion, which is a significant barrier to fintech adoption. Strategies should be developed to encourage adoption by addressing these issues. Third, expanding product coverage with regard to the acceptance of digital transactions and increasing the number of stores that accept ePayment services are critical factors in improving the adoption and usage of such services. Fourth, financial institutions and fintech firms should consider the importance of time and cost when designing payment processes to make them more convenient for users (Xu et al. 2022; Kou et al. 2021). They should also publicize less obvious risks and effective approaches to deal with them to promote the adoption of ePayment services. Fintech firms may also need to design infrastructures to address risks quickly and efficiently (Kou et al. 2021). Finally, integrating financial literacy programs into promotional efforts may encourage risk-averse customers to use fintech services. By providing information on the advantages of fintech services over traditional services and how to manage potential risks, users can make informed decisions about adopting ePayment services.

This study has valuable implications for policymakers seeking to promote the adoption of ePayment services. First, the findings demonstrate a positive relationship between financial literacy and fintech adoption. Therefore, policies to improve financial literacy, including financial and digital financial education programs, should be implemented in schools and workplaces. These efforts are critical because financial education indirectly affects fintech adoption and usage through financial literacy (Yoshino et al. 2017). Second, because fintech adoption and use vary based on factors such as age, sex, education, occupation, and income, a one-size-fits-all approach to promoting the uptake of fintech services may not be effective. Policymakers should target different groups using tailored strategies to address their specific needs. Finally, this study provides insights into how to design appropriate policies to promote a cashless economy in a cash-loving society such as Japan. Policymakers should consider the factors that motivate people to continue using cash and design policies to address these issues. For example, policies could be implemented to incentivize merchants to adopt ePayment systems and encourage consumers to use them. Such initiatives could include discounts or other incentives for stores and businesses to use ePayment systems. In summary, this study’s findings demonstrate the importance of financial literacy, targeted policies, and thoughtful strategies to promote the uptake of fintech services and the adoption of a cashless economy. Policymakers should consider these factors when designing policies and programs to encourage the use of fintech services.

### Limitations and future research

Although our study sheds light on the relationship between financial literacy and ePayment adoption, it has some limitations. First, although our measure of financial literacy is more comprehensive than most current measures in the literature, it lacks indicators that could be more relevant to digital finance, such as knowledge of digital risks and ways to control them. Second, owing to data limitations, we could not explore the effects of the COVID-19 pandemic on the relationship between financial literacy and ePayment services. Third, we could not examine the relationship between financial literacy and other aspects of fintech besides payment methods. Fourth, our study was limited to only two behavioral traits—risk aversion and herd behavior—and we could not construct more comprehensive indicators to reflect individuals’ behavioral traits. Therefore, careful interpretation of our results is required to determine the effects of behavioral factors. Finally, our study focused on the causal effects of financial literacy on ePayment adoption and usage in Japan. Future research can extend it to answer other interesting questions, such as how financial literacy affects payment choice or how cash preference and credit card ownership affect people’s adoption and usage of ePayment instruments.

### Supplementary Information

**Additional file 1: Appendix 1** Financial literacy questions. **Appendix 2:** Fintech adoption by gender, age, occupation, education and income

## Data Availability

The original data for this study is not allowed to share by the authors. However, the interested parties could contact the Bank of Japan’s Financial Services Agency to have a copy of the database.

## Appendix 1

See the Table 8.

**Table 8:** First stage estimation results Source: Authors’ estimation

	(1)	(2)	(3)	(4)
	Financial literacy	Finacial literacy * Risk Aversion	Financial literacy	Finacial literacy * Herd behavior
IV: Average financial literacy	0.069***	− 0.073***	0.098***	− 0.006
	[0.009]	[0.014]	[0.008]	[0.004]
IV: Awareness of changes adult age	0.617***	1.058***	0.618***	0.113***
	[0.014]	[0.027]	[0.014]	[0.007]
IV: Average financial literacy * Risk aversion	0.036***	0.289***		
	[0.006]	[0.008]		
IV: Average financial literacy * Herd behavior			− 0.009	0.121***
			[0.006]	[0.006]
Risk aversion	− 0.747***	− 3.849***	− 0.234***	− 0.041***
	[0.081]	[0.113]	[0.012]	[0.006]
Herd behavior	− 0.183***	− 0.232***	− 0.065	− 1.888***
	[0.014]	[0.025]	[0.089]	[0.089]
Limited acceptance (of stores)	0.133***	0.194***	0.132***	0.020***
	[0.012]	[0.021]	[0.012]	[0.006]
Long time to settle payment	− 0.171***	− 0.256***	− 0.170***	− 0.015*
	[0.018]	[0.032]	[0.018]	[0.009]
Not adequately secure private information	0.228***	0.387***	0.227***	0.023***
	[0.012]	[0.022]	[0.012]	[0.006]
Lack of tools to prevent overuse	0.070***	0.129***	0.069***	0.034***
	[0.015]	[0.028]	[0.015]	[0.007]
Fully satisfied with cash	− 0.218***	− 0.384***	− 0.219***	− 0.014**
	[0.015]	[0.028]	[0.015]	[0.007]
Male	0.058***	0.088***	0.062***	0.011*
	[0.013]	[0.024]	[0.013]	[0.006]
Age group (reference group: ≤ 30)				
30–40 years	− 0.036	− 0.056	− 0.033	− 0.012
	[0.025]	[0.047]	[0.025]	[0.013]
40–50	− 0.043	− 0.065	− 0.041	− 0.012
	[0.030]	[0.056]	[0.031]	[0.016]
50–60	− 0.055	− 0.029	− 0.052	− 0.019
	[0.040]	[0.073]	[0.040]	[0.020]
60–70	− 0.062	− 0.025	− 0.058	− 0.013
	[0.046]	[0.086]	[0.046]	[0.023]
≥ 70	− 0.068	− 0.029	− 0.065	− 0.022
	[0.048]	[0.088]	[0.048]	[0.023]
Education (reference group: primary and junior high school)				
High school	0.229***	0.415***	0.229***	0.026
	[0.029]	[0.057]	[0.029]	[0.016]
Specialized college	0.214***	0.449***	0.214***	0.024
	[0.031]	[0.062]	[0.031]	[0.017]
Junior college/tech college	0.303***	0.567***	0.305***	0.025
	[0.032]	[0.062]	[0.032]	[0.017]
University	0.487***	0.822***	0.486***	0.070***
	[0.029]	[0.058]	[0.029]	[0.016]
Graduate school	0.662***	0.989***	0.664***	0.074***
	[0.038]	[0.073]	[0.038]	[0.020]
Income (JPY) (reference group: no income)				
< 2.5 ml	0.186***	0.247***	0.187***	0.023
	[0.032]	[0.062]	[0.032]	[0.018]
≥ 2.5 ml and < 5 ml	0.281***	0.433***	0.279***	0.045**
	[0.033]	[0.063]	[0.032]	[0.018]
≥ 5 ml and < 7.5 ml	0.374***	0.575***	0.372***	0.062***
	[0.034]	[0.065]	[0.034]	[0.018]
≥ 7.5 ml and < 10 ml	0.428***	0.647***	0.425***	0.065***
	[0.035]	[0.068]	[0.035]	[0.019]
≥ 10 ml and < 15 ml	0.417***	0.579***	0.414***	0.054***
	[0.038]	[0.072]	[0.038]	[0.021]
≥ 15 ml	0.357***	0.527***	0.354***	0.046*
	[0.050]	[0.089]	[0.050]	[0.024]
Don't report income	0.002	− 0.088	0.000	0.008
	[0.033]	[0.063]	[0.032]	[0.018]
Other covariates	Yes	Yes	Yes	Yes
Intercept	− 1.725***	− 0.953***	− 2.150***	− 0.048
	[0.112]	[0.184]	[0.095]	[0.045]
N	24,516	24,516	24,516	24,516

*, **, *** Indicate significance at the 10%, 5%, and 1% levels, respectively. Standard errors are in brackets. In our regressions, we also control for occupation, frequency of acquiring news, prefectural dummies. Columns 1 and 2 are the first stage regression results with two endogenous variables of financial literacy and the interaction between financial literacy and risk aversion while the columns 3 and 4 are the first stage regression results with two endogenous variables of financial literacy and the interaction between financial literacy and herd behavior

## Abbreviations

E-money: Electronic money
ePayment: Electronic payment
fintech: Financial technology
GDP: Gross domestic product
IV: Instrumental variables
OLS: Ordinary least square

## Footnotes

1 Electronic payments, or ePayment services, are cashless payment methods (i.e., physical cash or money is not involved in the payment process) (Ab Hamid and Cheng 2013; Chen et al. 2019; Jeffus et al. 2017). In this manuscript, we focus on the usage of ePayment services by consumers. Furthermore, although there are several ePayment services, including credit/debit cards, chip-based electronic money (e-money), and mobile payment apps, we focus on the latter types since they are regarded as more innovative and more likely to promote financial inclusion.

2 Chip-based electronic money involves using an electronic medium that stores value on a chip. The top-up value for electronic money is reflected in the chip-based electronic medium. Electronic money can be classified as registered or unregistered, depending on their record-keeping method. Chip-based electronic money is anticipated to offer faster and more convenient payment processing than traditional systems (Aggarwal et al. 2020; Arifin 2018).

3 According to the Bank of Japan (2022), e-money in prepaid IC cards was introduced as early as 2001 as a fare collection system for public transportation. This form of money has gradually become a payment method accepted in many stores and supermarkets. While Japanese consumers still prefer cash payments (Saito 2021), IC cards such as Suica and Pasmo are gaining popularity as payment options for transportation fares, in-store purchases, and online transactions. The statistics from the Bank of Japan (2022) show that (i) in 2021, around 5.74 billion transactions with e-money were recorded, with a total value of transactions amounted to around 5.97 trillion Japanese Yen; (ii) the number of issued pre-paid IC cards increased to 470.07 million from 447.86 million in 2020. Regarding mobile payment apps, recent data shows a growing trend in adopting mobile payment apps, especially apps with QR codes. In 2020, mobile payment apps accounted for almost 10% of the cashless instruments issued in Japan (Bank of Japan 2022). The transition of the QR Code Payment Market increased from 304.2 billion Yen in 2018 to 3870.2 billion Yen in 2020 (Yano Research Institute 2020).

4 Even though the UTAUT2 model is developed based on the hypothesis that behavioral intention is the underlying cause that determines the adoption of technological advancement, this model does not explicitly examine the role of behavior traits.

5 According to OECD/INFE (2016), financial literacy is related to (i) the ability to understand financial concepts so that financial literacy can be applied in making financial decisions; (ii) the ability to manage personal finances by using financial literacy to carry out financial activities such as recording income and expenses.; (iii) the ability to make the right decisions using financial literacy; and (iv) carrying out effective financial planning for future financial needs.

6 As mentioned in Footnote 3, only 10% of all new ePayment instruments issued in 2022 are mobile payment apps.

7 All the questions used to construct our index are taken from the Bank of Japan’s survey. As explained in "Data source" section, this index can be considered an extension of the index developed by the OECD/INFE (2016). In addition to the ten questions found in the OECD/INFE (2016) questionnaire, the Bank of Japan added questions to reflect their concept of financial literacy, which not only measures individuals’ financial understanding but also their knowledge of practical management of financial assets (Huston 2010). These added questions relating to literacy about debt and deposits, risk and insurance, and wealth building. For details, please refer to the Additional file 1: Appendix for the list of questions. Yoshino et al. (2017) provide a detailed list of questions to construct the financial literacy index and its features.

8 The survey does not have continuous data on age and income but rather on age and income groups.

9 The results from the probit regression are quantitatively similar to the results from the linear probability regression estimation. The results are available upon request.

10 We calculated the financial literacy index using principal component analysis as a robustness check. The estimation results using this index type are quantitatively similar to those presented in this section. The results are available upon request.
