# Supplementary material for: Financial literacy, behavioral traits, and ePayment adoption and usage in Japan
Source: Financ Innov. 2023 Jun 11;9(1):101. doi: 10.1186/s40854-023-00504-3 (PMC10257561; doi:10.1186/s40854-023-00504-3)
Supplement: Supplementary file 1 — Additional file 1: Appendix 1 Financial literacy questions. Appendix 2: Fintech adoption by gender, age, occupation, education and income [file 40854_2023_504_MOESM1_ESM.docx]

**FINANCIAL LITERACY AND FINTECH ADOPTION IN JAPAN**

**Online Appendix 1: Financial literacy questions**

| Category | Questions and answers |  |
| --- | --- | --- |
| Knowledge/Basics of transaction | Q1. Which of the following is inappropriate as an action to take when concluding a contract? Choose only one answer |  |
|  | a. Reconsidering whether the contract is truly necessary | |
|  | b. Checking whether cancellation of the contract is possible and whether a penalty is charged for doing so | |
|  | c. Concluding a contract based on a detailed explanation from the service provider, and carefully reading the contract document later |  |
|  | d. Seeking advice from a third party as needed when concluding a contract |  |
|  | e. Do not know |  |
|  | Q2 Which of the following is inappropriate as a behavior to avoid being involved in financial trouble? Choose only one answer. |  |
|  | a. Avoiding disclosing your personal information as much as possible |  |
|  | b. Making an effort to acquire financial and economic knowledge |  |
|  | c. Trusting and leaving the entire matter to the service provider when it is difficult to make a decision |  |
|  | d. Checking the user reviews of the product you are planning to purchase |  |
|  | e. Do not know |  |
|  | Q3. Which of the following is inappropriate as an action related to Internet transactions? Choose only one answer. |  |
|  | a. I updated the security software to the latest version |  |
|  | b. I received an e-mail, but I did not open it since it was sent from an unknown address |  |
|  | c. I made a bank transfer by using a computer at an Internet café |  |
|  | d. I checked many times to make sure that the information I entered had no errors |  |
|  | e. Do not know |  |
| Basic knowledge of economics and finance | Q4. Suppose you put 1 million yen into a savings account with a guaranteed interest rate of 2% per year. If no further deposits or withdrawals are made, how much would be in the account after 1 year, once the interest payment is made? Disregard tax deductions. Answer with a whole number. |  |
|  | Q5. Compound interest: Then, how much would be in the account after 5 years? Disregard tax deductions. Choose only one answer. |  |
|  | a. More than 1.1 million yen |  |
|  | b. Just 1.1 million yen |  |
|  | c. Less than 1.1 million yen |  |
|  | d. Impossible to tell from the information given |  |
|  | e. Do not know |  |
|  | Q6. Imagine that the interest rate on your savings account was 1% per year and inflation was 2% per year. After 1 year, how much would you be able to buy with the money in this account? Choose only one answer |  |
|  | a, More than today |  |
|  | b. Exactly the same |  |
|  | c, Less than today |  |
|  | d. Do not know |  |
|  | Q7. High inflation means that the cost of living is increasing rapidly |  |
|  | a. Correct |  |
|  | b. Wrong |  |
|  | c. Do not know |  |
|  | Q8. If interest rates rise, what will typically happen to bond prices? Choose only one answer. |  |
|  | a. Go up |  |
|  | b. Go down |  |
|  | c, It does not change |  |
|  | d. There is no relationship between bond prices and interest rates |  |
|  | e. Do not know |  |
|  | Q9: Which of the following is appropriate as an action to take when investing (making deposits, etc.) or borrowing funds at a time of interest rate rise? Choose only one answer. |  |
|  | a. Investing and borrowing at fixed interest rates |  |
|  | b. Investing at a fixed interest rate and borrowing at a floating interest rate |  |
|  | c. Investing at a floating interest rate and borrowing at a fixed interest rate |  |
|  | d. Investing and borrowing at floating interest rates |  |
|  | e. Do not know |  |
| Basic knowledge of loans and credit | Q10. When compared, a 15-year mortgage typically requires higher monthly payments than a 30-year loan, but the total interest paid over the life of the loan will be less |  |
|  | a. Correct |  |
|  | b. Wrong |  |
|  | c. Do not know |  |
|  | Q11. Which of the following statements on mortgages is appropriate? Choose only one answer |  |
|  | a. It is far less costly to continue living in a rented house for your whole life than buying a house with a loan |  |
|  | b. Mortgages can be repaid by either the equal payment method or the equal principal payment method, but the total repayment is the same for both methods |  |
|  | c. Mortgages are offered with either a floating interest rate or a fixed interest rate, and those with a fixed interest rate are always more advantageous than those with a floating interest rate |  |
|  | d. In order to decrease the total mortgage repayment, it is effective to prepare as much down payment as possible and make advanced repayments to the extent possible |  |
|  | e. Do not know |  |
|  | Q12. Suppose you owe 100,000 yen on a loan and the interest rate you are charged is 20% per year compounded annually. If you didn't pay anything off, at this interest rate, how many years would it take for the amount you owe to double? Choose only one answer. |  |
|  | a. Less than 2 years |  |
|  | b. At least 2 years but less than 5 years |  |
|  | c. At least 5 years but less than 10 years |  |
|  | d. At least 10 years |  |
|  | e. Do not know |  |
| Basic knowledge of insurance | Q13: Which of the following statements on the basic function of insurance is appropriate? Choose only one answer. |  |
|  | a. Insurance is effective when a risk occurs with high frequency, causing a large risk |  |
|  | b. Insurance is effective when a risk occurs with low frequency, causing a large risk |  |
|  | c. Insurance is effective when a risk occurs with high frequency, causing a small risk |  |
|  | d. Insurance is effective when a risk occurs with low frequency, causing a small risk |  |
|  | e. Do not know |  |
|  | Q14. When a 50-year-old man reviews his life insurance policy (whole life insurance) after his children have become financially independent, which of the following statements is appropriate? Suppose that other circumstances have not changed. Choose only one answer. |  |
|  | a. He should consider increasing the death benefit |  |
|  | b. He should consider decreasing the death benefit |  |
|  | c. There is no need to review the policy in particular |  |
|  | d. Do nott know |  |
|  | Q15. Which of the following statements on insurance is inappropriate? Choose only one answer. |  |
|  | a. You need to pay national pension contributions if you are aged 20 or over, even if you are a student |  |
|  | b. The damage caused by an automobile accident will be fully covered by the automobile liability insurance |  |
|  | c. You should review the necessity of life insurance and the amount of coverage of insurance according to changes in circumstances of family members and yourself |  |
|  | d. Health insurance may not cover pre-existing medical conditions that you had before purchasing the insurance policy |  |
|  | e. Do not know |  |
| Basic knowledge of wealth building | Q16. An investment with a high return is likely to be high risk |  |
|  | a. Correct |  |
|  | b. Wrong |  |
|  | c. Do not know |  |
|  | Q17. Buying a single company's stock usually provides a safer return than a stock mutual fund |  |
|  | a. Correct |  |
|  | b. Wrong |  |
|  | c. Do not know |  |
|  | Q18. Which of the following statements on the types of deposits protected up to 10 million yen under Japan's deposit insurance system is appropriate? Choose only one answer |  |
|  | a. Only ordinary deposits are protected |  |
|  | b. Ordinary deposits and time deposits are protected |  |
|  | c. All types of deposits including ordinary deposits, time deposits, and foreign currency deposits are protected |  |
| Family Budget Management | Q19. Which of the following statements on household behavior is inappropriate? Choose only one answer. |  |
|  | a. Managing income and expenditure by keeping a household account book or the like |  |
|  | b. Deciding on expenditure after considering whether it is truly necessary and whether there is enough income |  |
|  | c. Saving some money out of income by transferring a fixed amount of income into a savings account or the like |  |
|  | d. Frequently using installment payment plans of credit cards in order to defer payment |  |
|  | e. Don't know |  |
|  | Q20. Which of the following statements on family budget management and credit cards is inappropriate? Choose only one answer. |  |
|  | a. Using credit cards in a well-planned manner according to income |  |
|  | b. Any unsettled credit card payment is practically a debt |  |
|  | c. A credit card fee (interest) is charged for revolving payments but not for installment payments |  |
|  | d. Failure to pay the credit card charge may cause credit card transactions to be declined |  |
|  | e. Don't know |  |
| Life planning | Q21. Taro and Hanako are the same age. At age 25 Hanako began saving 100,000 yen per year and continued to save the same amount annually thereafter. Meanwhile, Taro did not save money at age 25, but began saving 200,000 yen per year at age 50. When they are aged 75, which of them will have more money saved? Choose only one answer. |  |
|  | a. They would each have the same amount because they put away exactly the same amount |  |
|  | b. Taro, because he saved more each year |  |
|  | c. Hanako, because she has put away more money |  |
|  | d. Hanako, because her money has grown for a longer time at compound interest |  |
|  | e. Do not know |  |
|  | Q22: What are the so-called three major expenses in life? Choose only one answer. |  |
|  | a. Living expenses for your lifetime, children's educational expenses, and your medical expenses |  |
|  | b. Children's educational expenses, costs of buying a house, and living expenses for your retirement |  |
|  | c. Costs of buying a house, your medical expenses, and costs of nursing care for your parents |  |
|  | d. Do not know |  |
| Use of outside expertise | Q23. Which of the following is inappropriate as behavior or attitude when determining whether to purchase an unfamiliar financial product? Choose only one answer. |  |
|  | a. Collecting information to make sure that the product is not frequently causing trouble and no warning has been issued by a public institution |  |
|  | b. Collecting information from the Internet, books, and several sellers and comparing the product with other products |  |
|  | c. Consulting with an institution, agency, etc., that provides information from a neutral standpoint and receiving advice |  |
|  | d. Purchasing the product if the seller tells you that you can expect a high return |  |
|  | e. Do not know |  |
|  | Q24. Which of the following is appropriate as an action to take when considering purchase of a financial product with a complicated structure? Choose only one answer. |  |
|  | a. Purchasing the product if it is selling well, even if you do not understand its structure clearly |  |
|  | b. Purchasing the product if you can trust the financial institution providing the product, even if you do not understand its structure clearly |  |
|  | c. Purchasing the product if you can expect a high return, even if you do not understand its structure clearly |  |
|  | d. Purchasing the product if you understand its structure and find no problem |  |
|  | e. Do not know |  |
|  | Q25. Which of the following is inappropriate as a consultant office or a system to be used when trouble occurs in relation to a contract for a financial product? Choose only one answer. |  |
|  | a. Consumer center |  |
|  | b. Financial alternative dispute resolution (ADR) system |  |
|  | c. Rating company |  |
|  | d. Attorney at law |  |

Source: CCFSI (2019).

***Online Appendix 2: Fintech adoption by gender, age, occupation, education and income***

|  | Use electronic money | Use electronic money almost every day | Use payment apps (mobile) | Use payment apps (mobile) almost every day | Holding crypto assets |
| --- | --- | --- | --- | --- | --- |
| Whole sample | 35.8% | 10.3% | 8.0% | 2.1% | 7.8% |
| Gender |  |  |  |  |  |
| Male | 36.7% | 12.4% | 11.0% | 3.2% | 11.0% |
| Female | 34.9% | 8.3% | 5.0% | 1.0% | 4.6% |
| Age group |  |  |  |  |  |
| <=30 | 31.9% | 11.7% | 11.8% | 3.2% | 11.9% |
| 30>=40 | 36.9% | 11.9% | 12.3% | 3.2% | 11.6% |
| 40>=50 | 39.3% | 12.1% | 9.8% | 2.5% | 9.0% |
| 50>=60 | 41.6% | 12.2% | 7.6% | 2.1% | 7.2% |
| 60>=70 | 36.3% | 8.0% | 4.3% | 1.2% | 4.4% |
| >70 | 26.7% | 5.6% | 2.0% | 0.5% | 2.8% |
| Education level |  |  |  |  |  |
| Primary/secondary/others | 23.6% | 6.6% | 5.9% | 1.9% | 5.5% |
| High school | 29.7% | 7.9% | 6.3% | 1.8% | 5.5% |
| Specialized college | 34.1% | 9.3% | 7.2% | 1.6% | 7.0% |
| Junior college/tech college | 36.2% | 8.2% | 5.2% | 0.9% | 5.5% |
| University | 41.0% | 12.8% | 10.0% | 2.7% | 10.1% |
| Graduate school | 48.4% | 17.4% | 13.9% | 3.9% | 14.4% |
| Occupation |  |  |  |  |  |
| Company employee | 41.9% | 15.5% | 12.8% | 3.8% | 12.3% |
| Gov’t employee | 44.1% | 13.1% | 12.6% | 3.2% | 13.2% |
| Teacher | 42.1% | 14.4% | 11.0% | 3.0% | 8.0% |
| Self-employed | 33.0% | 8.5% | 8.0% | 1.8% | 8.2% |
| Part-timer | 36.1% | 9.6% | 6.2% | 1.4% | 4.8% |
| Homemaker | 32.2% | 5.3% | 3.1% | 0.3% | 3.7% |
| Students | 36.0% | 14.1% | 10.8% | 2.7% | 11.1% |
| Unemployed/others | 26.3% | 5.1% | 3.6% | 1.0% | 4.1% |
| Yearly income (JPY) |  |  |  |  |  |
| Less than 5 mil. | 32.9% | 9.4% | 6.8% | 1.7% | 7.2% |
| From 5 mill. to 10 mil. | 41.8% | 12.4% | 10.2% | 2.6% | 10.1% |
| More than 10 mil. | 50.2% | 16.4% | 14.5% | 4.6% | 13.8% |
| Don't report | 28.6% | 7.3% | 5.1% | 1.3% | 3.5% |

Source: Authors’ calculation
